# HIV-1 Integrase Inhibitory Effects of Major Compounds Present in CareVid™: An Anti-HIV Multi-Herbal Remedy

**DOI:** 10.3390/life12030417

**Published:** 2022-03-12

**Authors:** Winnie Rotich, Eduard Mas-Claret, Nicholas Sadgrove, Anastasia Guantai, Guillermo F. Padilla-González, Moses K. Langat

**Affiliations:** 1 Department of Pharmacy, Sigowet-Soin Sub-County Hospital, Kericho County, P.O. Box 112, Kericho 20200, Kenya; winnierotichpharmacist@sigowethospital.org; 2 Royal Botanic Gardens Kew, Kew Green, Richmond, Surrey TW9 3AE, UK; e.mas-claret@kew.org (E.M.-C.); n.sadgrove@kew.org (N.S.); f.padilla@kew.org (G.F.P.-G.); 3 Department of Pharmacology and Pharmacognosy, School of Pharmacy, University of Nairobi, P.O. Box 19676-00202, Nairobi 00100, Kenya; anguantai@uonbi.ac.ke

**Keywords:** CareVid^TM^, HIV-1 integrase, ellagic acid, pellitorine, urolithin A and urolithin B, molecular docking

## Abstract

In our continued study on the anti-HIV activity of compounds present in CareVid^TM^, we report the HIV-1 integrase ((HIV-1 IN) inhibitory effects of pellitorine (**1**), oleuropein (**2**), magnoflorine (**3**), crotepoxide (**4**), *ent*-kaurane-16β,17-diol (**5**), crotocorylifuran (**6**), lupeol (**7**), betulin (**8**), and ellagic acid (**9**) in an in vitro enzyme assay, and in an in silico study. Ellagic acid, pellitorine, lupeol, and betulin showed an in vitro percentage inhibition against HIV-1 IN of 21.1%, 19.0%, 18.5%, and 16.8%, respectively, at a standard concentration of 25 μg/mL. However, from a pharmacokinetic perspective, ellagic acid has poor bioavailability, due to rapid elimination in metabolism in the gut microbiome. It was postulated that known gut catabolites of ellagic acid, urolithin A (**10**) and urolithin B (**11**) could be more promising candidates in exploring the anti-HIV activity of ellagic acid-rich medicinal species consumed orally. On the contrary, urolithin A and urolithin B demonstrated lower activity with comparison to ellagic acid. The binding affinity of compounds **1**–**9**, urolithin A, and urolithin B against the catalytic domain of HIV-1 IN was also explored by in silico methods. Docking studies showed oleuropein as the best candidate, with a predicted energy of binding of ΔG −5.81 kcal/mol, while ellagic acid showed moderate predicted inhibition (ΔG −4.38 kcal/mol) caused by the interaction between the carbonyl and the key Mg^2+^ ion in the active site.

## 1. Introduction

In the current study, we report the in silico and in vitro HIV-1 integrase (HIV-1 IN) effects of compounds isolated from CareVid^TM^ tea powder, pellitorine (**1**), oleuropein (**2**), magnoflorine (**3**), crotepoxide (**4**), *ent*-kaurane-16β,17-diol (**5**), crotocorylifuran (**6**), lupeol (**7**), betulin (**8**), and ellagic acid (**9**). CareVid^TM^ is a multi-herbal remedy that is widely used in Kericho, south west Kenya, as an immune booster and a health tonic by HIV-positive patients [1]. From the results, ellagic acid was modestly active; hence, we also studied the in silico and in vitro HIV-1 IN effects of known gut catabolites of ellagic acid, urolithin A (**10**) and urolithin B (**11**). 

CareVid^TM^ is a patented herbal powder that is made from the roots, bark, and whole plant of 14 African medicinal plants [2,3], *Acacia nilotica* (L.) Willd. ex Delile (currently, *Vachelia nilotica* (L.) P.J.H Hurter and Mabb.), *Adenia gummifera* (Harv.) Harms, *Anthocleista grandiflora* Gilg, *Asparagus africanus* Lam., *Bersama abyssinica* Fresen., *Clematis hirsuta* Guill. and Perr., *Croton macrostachyus* Hochst. ex Delile, *Clutia robusta* Pax (accepted as *Clutia kilimandscharica* Engl.), *Dovyalis abyssinica* (A. Rich.) Warb, *Ekebergia capensis* Sparm., *Periploca linearifolia* Quart.-Dill. and A. Rich., *Plantago palmata* Hook.f., *Prunus africana* Hook.f. Kalkman, and *Rhamnus prinoides* L’Her [2,3]. The ethnomedicinal uses on HIV and other related diseases of the 14 plants have been summarized in Rotich et al., 2021 [1,4], whereas a detailed ethnomedicinal uses on HIV-unrelated diseases are presented in an unpublished review [4]. 

The powdered herbal product, CareVid^TM^, is infused in hot water and consumed as a concoction. People living with HIV who are non-compliant with their antiretroviral treatments due to cultural and financial barriers consume the tea as an alternative. Through visitation to their clinics, many have reported increased CD4+ cell counts [2,3]. 

In our earlier chemical study of CareVid^TM^, we reported that it is characterised by the major compounds pellitorine (**1**), oleuropein (**2**), magnoflorine (**3**), crotepoxide (**4**), *ent*-kaurane-16β,17-diol (**5**), crotocorylifuran (**6**), lupeol (**7**), betulin (**8**), and ellagic acid (**9**) [1]. In an unpublished review [4], the presence of oleuropein (**2**) in the tea was linked to *D. abyssinica*. Crotepoxide (**4**), *ent*-kaurane-16β,17-diol (**5**), crotocorylifuran (**6**), and betulin (**8**) are derived from *C. macrostachyus*, and lupeol (**7**) is from two biota, i.e., *C. macrostachyus* and *C. hirsuta*. Lastly, ellagic acid (**9**) is derived from both *V. nilotica* and *B. abyssinica*. 

Out of all these compounds, only ellagic acid has been previously tested for in vitro HIV-1 enzyme inhibition. Ellagic acid has previously shown moderate inhibition of the protease enzyme and it also interferes with the activity of LEDGF/p75, a protein that acts as a docking factor or receptor for tethering HIV-IN to chromatin [5]. While ellagic acid strongly inhibited the tethering step to integration, the current study focuses only on the integrase enzyme.

In the search for new anti-HIV-1 agents, HIV-IN represents a potential target for natural products [6]. HIV-1 IN is one of the enzymes responsible for the integration of viral DNA into the host DNA, catalysing 3′-processing and DNA strand transfer reactions [7]. Initially, the viral DNA is cleaved by HIV-1 IN at a CA dinucleotide, at the 3′ end, to leave the two-nucleotide overhanging, in a step that is known as processing. This is followed by transportation of the protein–DNA complex into the nucleus. The host DNA is cleaved to leave a 5′ overhang of five bases and the 3′ ends of the viral DNA are covalently linked to the 5′ end of the host DNA [7]. Finally, the 5-bases gap between the 5′ end of viral DNA and the 3′ end of host DNA is filled in by host cell enzymes [8]. 

In mainstream antiretroviral (ARV) therapy, blocking of HIV-1 integration is achieved using a class of drugs called integrase strand transfer inhibitors (INSTIs). INSTIs bind to an intermediate in DNA integration called the intasome, in which a pair of viral DNA ends are in a synaptic complex with the tetramer of integrase [6]. Since 2007, five INSTIs have been introduced: raltegravir, elvitegravir, dolutegravir, bictegravir, and cabotegravir [9]. However, these drugs have limited clinical benefit because long-term treatments may lead to the emergence of drug resistance and side effects [10]. Therefore, finding alternative agents that take the pressure away from these more efficient INSTIs will slow the progress of resistance development [11]. 

In the development of new INSTIs, the use of in vitro assay systems and recombinant HIV-1 IN has been used as a preliminary discovery tool, prior to pharmacokinetic evaluation. Amongst others, Tewtrakul et al. [12] have demonstrated that medicinal plants possess anti-HIV-1 IN inhibitory effects. For example, thalassiolin A and curcumin are examples of natural products that have showed anti-HIV-1 IN effects [13]. 

In the current study, we recognised that significant amounts of ellagic acid is produced in human digestion by acid hydrolysis of ellagitannins of CareVid^TM^ in the stomach. Thereafter, ellagic acid is incapable of achieving systemic concentrations high enough to exert the effects of an in vitro outcome. This is because ellagic acid is catabolised in the microbial digestion into simpler urolithins. Because these tannin-rich barks and roots are a major component of the CareVid^TM^ tea, candidates are likely to have high quantities of circulation urolithins. Therefore, we studied the inhibitory effects of HIV-1 IN by urolithin A and urolithin B.

## 2. Materials and Methods

### 2.1. Chemicals

**Figure 1:**
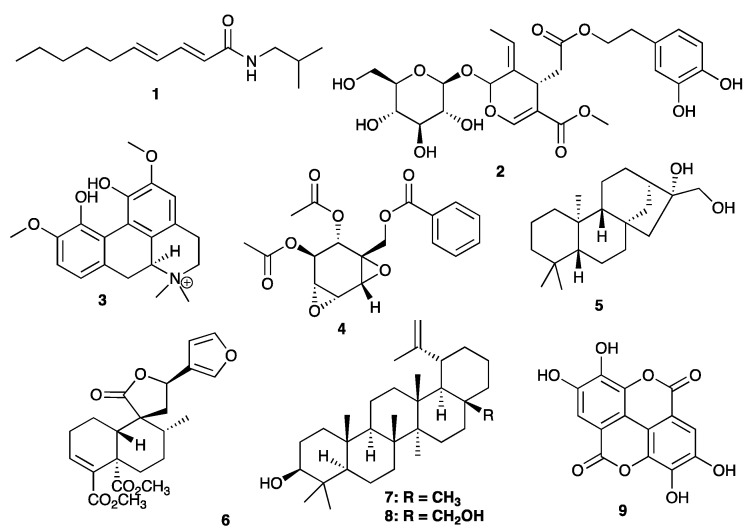
Compounds present in CareVid^TM^ tested for Anti-HIV 1 IN inhibition.

The nine compounds (**1**–**9**) were determined using HPLC-MS to be present in CareVid^TM^ as described previously [1]. Extracts were prepared initially from 20 g each of CareVid^TM^ Tea powder. The extracts were methylene chloride, ethanol, methanol, water, acidified water (0.1 M HCl), and steeping in hot water (>90 °C). Metabolic profiling of the extracts was performed by UHPLC-UV-HRMS/MS on a Vanquish UHPLC system (Thermo Scientific, Waltham, MA, USA) coupled to a 100 Hz photodiode array detector (PDA) and an Orbitrap Fusion Tribrid (Thermo Scientific) high-resolution tandem mass spectrometer (Thermo Scientific, Waltham, MA, USA). A detailed protocol that was followed is described in Rotich et al., 2021 (Figure 1). For the assay, pure standards of pellitorine (**1**), oleuropein (**2**) and ellagic acid (**9**), urolithin A (**10**), and urolithin B (**11**) were bought from Sigma Aldrich, whereas pure standards of magnoflorine (**3**) earlier isolated from the seed cake of *Croton megalocarpus*, crotocorylifuran (**6**) earlier isolated from *Croton megalocarpoides,* crotepoxide (**4**) earlier isolated from *Croton alienus*, *ent*-kaurane-16β,17-diol (**5**) earlier isolated from *Croton haumanianus*, and lupeol (**7**) and betulin (**8**) isolated in substantial quantities from CareVid^TM^ were obtained in the Jodrell Laboratory, Kew, UK.

### 2.2. In Vitro HIV-1 Integrase 

The XpressBio HIV-1 Integrase Assay Kit (EZ-1700, Kit Lot K1029), provided by Express Biotech International, was used to measure the inhibitory effects of the different compounds on HIV-1 IN activity following the manufacturer’s instructions. Briefly, Streptavidin coated 96-well plates were coated with a double-stranded HIV-1 LTR U5 donor substrate (DS) oligonucleotide containing an end-labelled biotin. Full-length recombinant HIV-1 IN protein was then loaded onto the DS DNA substrate. Compounds and sodium azide (positive control) were added to the enzyme reaction and then a different double-stranded target substrate (TS) DNA containing a 3′-end modification was added to the reaction mixture. The HIV-1 IN cleaves the terminal two bases from the exposed 3′-end of the HIV-1 LTR DS DNA and then catalyses a strand-transfer recombination reaction to integrate the DS DNA into the TS DNA. The products of the reaction were detected calorimetrically using an HRP-labelled antibody directed against the TS 3′-end modification and the absorbance due to the HRP antibody–TMB peroxidase substrate reaction was measured at 450 nm. Compounds were dissolved in dimethyl sulfoxide to form a 20 mg/mL solution that was diluted with nuclease free water to double the desired starting concentration. A final concentration of 25 μg/mL was used for all compounds. For sodium azide, a concentration range of 0.05–1.5% was used to calculate its IC_50_ value (See Supporting Information, Figure S10). All samples were assayed in triplicate and results presented as the averages. All samples were assayed in triplicate and results are provided as mean ± standard deviation (SD). Statistical analysis of the data was carried out by one way ANOVA (Graph Pad Prism 9.3.1 Software). A value of *p* < 0.05 was considered to be significant.

### 2.3. Docking Methodology

The docking studies were performed on MOE2015 software package HIV-1 IN catalytic domain complexed with inhibitor 5CITEP (PDB ID: 1QS4). The protein was prepared by first removing all water molecules. Then, missing side chains were reconstructed, hydrogens were added, and the structures were protonated. The ligands were energy minimised using molecular mechanics forcefield MMFF94x. To validate the docking protocol used, known inhibitor 5CITEP was removed from their corresponding binding pockets and redocked. The Root Mean Square Deviation (RMSD) value from the known co-crystallized conformation was 0.8212 Å. The compounds were docked using MOE2015 with triangle matcher, scoring by London dG, 100 poses as placement method and rigid receptor, GBVI/WSA dG 5 poses as refinement method in all targets, and the method was then repeated using induced fit as the refinement method. Both methods were repeated in three independent runs. The lowest scoring affinity pose in each ligand was used to study the ligand interactions [1]. 

## 3. Results

### 3.1. Compounds

The nine compounds (**1**–**9**) were determined to be present in CareVid^TM^ using HPLC-MS (Figure 1). Pellitorine (**1**) is a fatty amide previously reported from the roots of *Piper nigrum* [14]. Oleuropein (**2**) is a phenylethanoid, a type of secoiridoid phenolic compound found in the olive leaf, plants of Oleaceae, Gentianaceae, and Cornaleae, and is a major constituent of extra virgin oil [15]. Magnoflorine (**3**) is a quaternary aporphine alkaloid that is isolated from some commonly used herbal medicines (e.g., *Sinomenium acutum* (Thunb.) Rehder and E.H.Wilson and *Coptis chinensis* Franch.) [16]. Compounds **1**, **2,** and **3** have not been previously reported from the 14 plants that are used to make CareVid^TM^ [1]. Crotepoxide (**4**) is a cyclohexane diepoxide derivative [17], *ent*-kaurane-16β,17-diol (**5**) is a kaurenoid diterpenoid and crotocorylifuran (**6**) is a clerodane diterpenoid, and compounds **4**, **5**, and **6** have been reported from *Croton macrostachyus* [17,18], one of the 14 plants used to prepare CareVid^TM^. Lupeol (**7**) and betulin (**8**) are lupane type triterpenoids and both are widespread in the plant kingdom and have been previously reported from many plants that are used to prepare CareVid^TM^ [4]. Ellagic acid (**9**) is an organic heterotetracyclic compound resulting from the formal dimerisation of gallic acid by oxidative aromatic coupling with intramolecular lactonisation of both carboxylic acid groups of the resulting biaryl [19].

**Figure 2:**
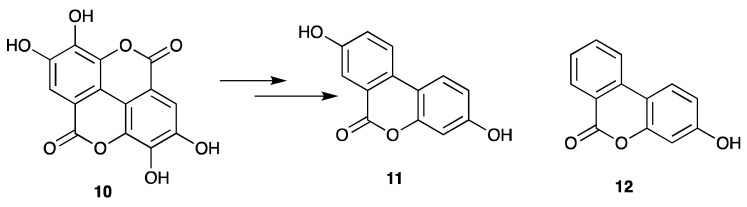
Structure of ellagic acid (**9**) present in CareVid^TM^ and its microbial catabolites, urolithin A and urolithin B [20].

Urolithin A (**10**) and urolithin B (**11**) are intestinal microbial metabolites produced from ellagic acid-containing products such as foods, walnuts, strawberries, and pomegranates (Figure 2). It is thought that these metabolites are more available than ellagic acid, and, hence, they can contribute significantly to the beneficial properties attributed to ellagic acid [20].

### 3.2. HIV-1 Integrase Catalytic Activity Inhibition

Compounds (**1**–**9**) that are present in CareVid^TM^ were tested in vitro for their ability to inhibit the enzyme HIV-1 IN (Table 1). Ellagic acid, pellitorine, lupeol, and betulin showed moderate inhibition, with values of 21.1%, 19.0%, 18.5%, and 16.8% relative to control (HIV-1 IN alone), respectively, at a concentration of 25 μg/mL. 

### 3.3. Molecular Docking

Compounds **1**–**11** tested in vitro were docked to the catalytic domain of HIV-1 IN complexed with 5CITEP (PDB ID: 1QS4) (Table 2; Tables S1 and S2). Both a rigid receptor and induced fit refinement models were used with similar results. Oleuropein (**2**) gave the best predicted results for free energy of binding (average ΔG −5.81 kcal/mol) (Figures S2–S9).

## 4. Discussion

In this study we describe in vitro and in silico inhibitory effects of compounds present in CareVid^TM^ against the HIV-1 IN activity. As previously described by Pflieger et al., 2013, natural products including phenolics exhibit inhibitory effects against HIV-1 IN enzyme [21]. Therefore, we discuss the inhibitory effects of pellitorine (**1**), oleuropein (**2**), magnoflorine (**3**), crotepoxide (**4**), *ent*-kaurane-16β,17-diol (**5**), crotocorylifuran (**6**), lupeol (**7**), betulin (**8**), and ellagic acid (**9**) and gut catabolites of ellagic acid, urolithin A (**10**) and urolithin B (**11**) at a single concentration of 25 μg/mL (Table 1). Ellagic acid, pellitorine, lupeol and betulin showed moderate inhibition, with values of 21.1%, 19.0%, 18.5%, and 16.8%. 

It is not surprising to have mild activity for ellagic acid at 25 µg.mL^−1^ (82.7 µM), as other studies have described in vitro viral inhibition with an IC_50_ of 8.7 µM, and LEDGF/p75 interference with an IC_50_ of 0.08 µM [22,23]. However, due to the poor pharmacokinetics of ellagic acid, the gut catabolites urolithin A (**10**) and urolithin B (**11**) are of greater relevance, but are nevertheless weakly active in inhibiting the HIV-1 IN enzyme, with 11.3% and 9.9% inhibition. We had hypothesised that urolithin A (**10**) and urolithin B (**11**) might be responsible for the anti-HIV activity attributed to ellagic acid, given the low stability of this metabolite in the human gut. Nevertheless, the in vitro assay results of urolithin A and urolithin B may be additive. However, with regards to the more clinically relevance concentration reported for LEDGF/p75 interference by ellagic acid, we encourage further examination using the urolithins using this system. 

Previously, pellitorine was tested for its inhibitory effect against HIV-1 reverse transcriptase [24]. In addition, lupeol (**7**) and betulin (**8**) are known HIV-1 IN inhibitors [11]. Furthermore, Huang et al., 2007 reported that oleuropein (**2**) inhibits HIV-1 IN, but we only observed modest inhibitory effects in the current study, inhibiting 4.7% at 25 μg/mL [25]. This is not explained but should be taken into consideration. 

Magnoflorine (**3**) was proposed as an anti-HIV compound by theoretical inhibition of HIV-1 reverse transcriptase [26], but this was not followed up by any in vitro test by the authors. However, in the current study, it weakly inhibited HIV-1 IN. Crotepoxide (**4**) has also been shown to inhibit HIV-1 reverse transcriptase, inhibiting 32% at 200 μg/mL [27], whereas *ent*-kaurane-16β,17-diol (**5**) and crotocorylifuran have not been described for their anti-HIV effects. However, a related derivative of 5, *ent*-16β,17-dihydroxykauran-19-oic acid is a known anti-HIV candidate [28]. 

**Figure 3:**
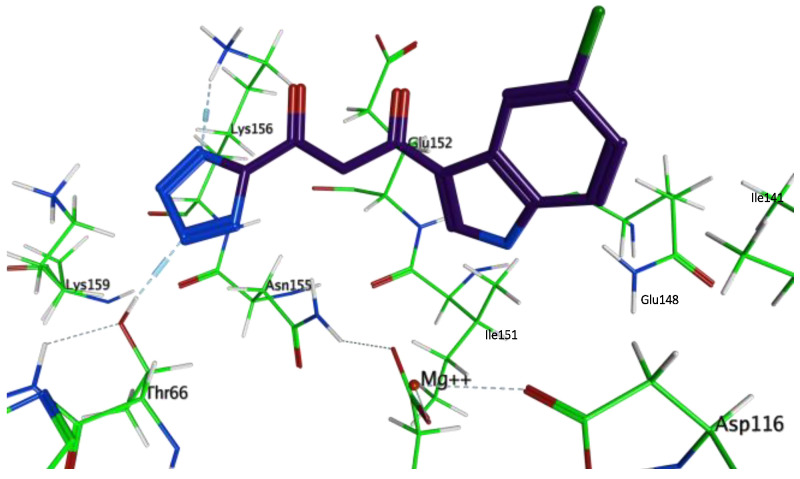
Inhibitor 5CITEP (purple) in complexation with the catalytic domain of HIV-1 IN (PDB ID: 1QS4).

The HIV-1 IN active site is comprised of two distinct cavities: one extending from Phe139 to Lys159 and the other including Asp64, Thr66, His67, Asp116, and Asn120, as well as Mg^2+^, which plays a key role in the 3′ processing activity of HIV-1 IN [29]. The Mg^2+^ ion is coordinated by a DDE catalytic triad motif, formed by three acidic residues in the active site (Asp64, Asp116, and Glu152). This motif is highly conserved among a superfamily of nucleases and polynucleotidyltransferases, and mutations introduced to residues forming the catalytic triad have been shown to greatly diminish HIV-1 IN activity [30]. Diketoacids (DKAs), known inhibitors of HIV-1 IN, act by coordinating the Mg^2+^ ion in the IN active site, resulting in a functional sequestration of the critical metal cofactor [10]. However, 1-(5-chloroindol-3-yl)-3-(tetrazolyl)-1,3-propadione enol (5CITEP), the first ligand for which a crystal structure was obtained in complexation with the catalytic domain of HIV-1 IN, did not present such contacts with either Asp64 or Asp116, and only showed an indirect, water-mediated contact with the bound Mg^2+^ ion [31]. As a consequence, it has been postulated that the exact mode of binding might have been influenced by crystal contacts [32]. Instead of interacting with the catalytic triad, it showed hydrogen bonding interactions between the tetrazole ring and Asn155, Thr66, Lys159, and Lys156, as well as hydrogen bonding between Gln148 and the nitrogen of the indole ring (Figure 3) [31]. 

**Figure 4:**
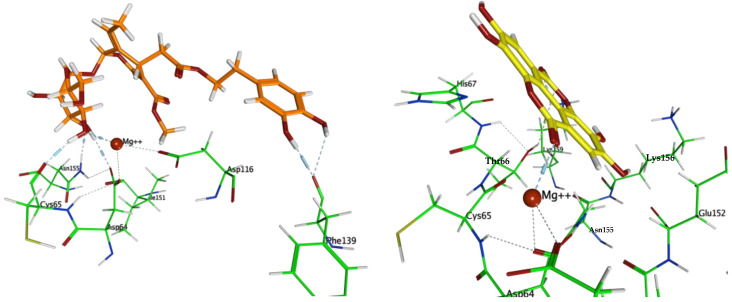
Highest scoring docked poses of top scoring ligand oleuropein **2** (orange) and ellagic acid **9** (yellow) in the catalytic domain of HIV-1 IN (PDB ID: 1QS4).

**Figure 5:**
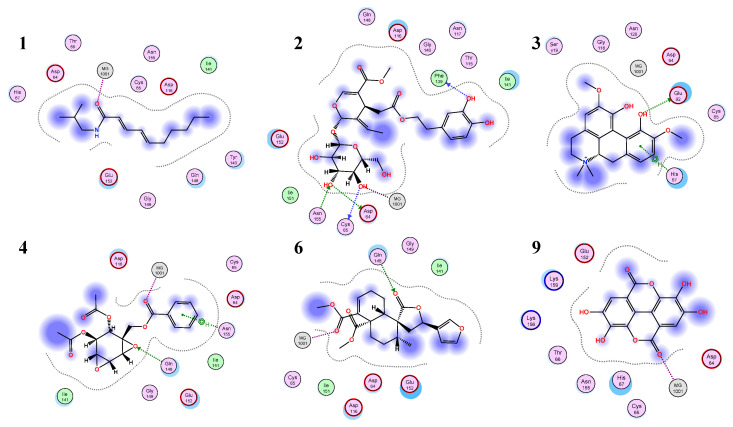
Ligand interactions between top scoring ligands and the active site of HIV-1 IN (PDB ID: 1QS4).

In this work, the eleven compounds tested in vitro were docked to the catalytic domain of HIV-1 IN complexed with 5CITEP (PDB ID: 1QS4) (Table 2; Figure S1). Both a rigid receptor and induced fit refinement models were used with similar results. Oleuropein (**2**) gave the best predicted results for free energy of binding (average ΔG −5.81 kcal/mol). The main interactions contributing to its predicted energy of binding are the hydrogen bonds between the phenolic hydroxyls and Phe139, as well as those between the glucose hydroxyls and key residues Asp64, Cys65, and Asn155 and the Mg^2+^ ion (Figure 4). Ellagic acid **9**, which gave the best results in terms of HIV-1 IN inhibition in vitro (21.1%), showed moderate inhibition in the docking study (average ΔG −4.38 kcal/mol), with the ion-dipole interaction with Mg^2+^ contributing favorably to its predicted free energy of binding (Figure 4). On the other hand, lupeol and betulin, which showed moderate inhibition in vitro, gave positive results of predicted free energy of binding in the three independent runs and, therefore, they were not included in the discussion. Their poor predicted energy of binding could be due to the large size of those candidates causing excessive steric clash with the residues in the active site, leading to a large increase in predicted free energy of binding in the in silico model. Ligand interactions for the best scoring ligands are displayed in Figure 5.

## 5. Conclusions

Compounds (**1**–**9**) that are present in CareVid^TM^ were tested in vitro for their ability to inhibit the enzyme HIV-1 IN (Table 1). Ellagic acid, pellitorine, lupeol, and betulin showed moderate inhibition, with values of 21.1%, 19.0%, 18.5%, and 16.8% relative to control (HIV-1 IN alone), respectively, at a concentration of 25 μg/mL. Docking studies showed oleuropein as the candidate with lowest predicted energy of binding (ΔG −5.81 kcal/mol), while ellagic acid showed moderate predicted inhibition (ΔG −4.38 kcal/mol) caused by the interaction between the carbonyl and the key Mg^2+^ ion in the active site. This study, on the effects against HIV-1 IN, is a continuation of the initial study of authentic reference compounds found in CareVid^TM^ against HIV-1 RT. Therefore, a future study on the effects against protease is necessary, and perhaps subjecting the compounds through whole cell assay. In addition, bioassay guided fractionation to identify minor components that may have inhibitory effects against HIV-1 enzymes may also be necessary.

## Figures and Tables

**Table 1:** % Activity relative to control (HIV-1 integrase) values obtained for the different compounds tested against HIV-1 Integrase. Compounds were assayed at a concentration of 25 μg/mL ^a^.

Treatment	% Activity rel. to Control	% Inhibition
HIV Integrase (no inhibition)	100	0
Pellitorine (**1**)	81.0 ± 9.1 *	19.0
Oleuropein (**2**)	95.3 ± 4.2	4.7
Magnoflorine (**3**)	88.0 ± 7.5	12.0
Crotepoxide (**4**)	91.0 ± 5.0	9.0
*Ent*-kaurane-16β,17-diol (**5**)	86.6 ± 2.1	13.4
Crotocorylifuran (**6**)	90.9 ± 5.0	9.1
Lupeol (**7**)	81.5 ± 1.2 *	18.5
Betulin (**8**)	83.2 ± 13.6	16.8
Ellagic acid (**9**)	78.9 ± 5.7 *	21.1
Urolithin a (**10**)	88.7 ± 5.4	11.3
Urolithin b (**11**)	90.1 ± 3.8	9.9

^a^ Results presented as mean ± standard deviation (n = 3). The data were analyzed with the one-way ANOVA, using Dunnett’s test with a significance level set at 0.05. * *p* < 0.05.

**Table 2:** Calculated free energy of ligands docked with HIV-1 IN.

	Free Energy of Binding (ΔG) kcal/mol	
Compound	Rigid Receptor	Induced Fit
5CITEP	−4.42	−4.18
Pellitorine (**1**)	−4.34	−4.22
Oleuropein (**2**)	−5.67	−5.94
Magnoflorine (**3**)	−4.24	−4.25
Crotepoxide (**4**)	−4.75	−4.96
*Ent*-kaurane-16β,17-diol (**5**)	>0	>0
Crotocorylifuran (**6**)	−4.33	−4.21
Lupeol (**7**)	>0	>0
Betulin (**8**)	>0	>0
Ellagic acid (**9**)	−4.16	−4.60
Urolithin A (**10**)	−3.95	−3.91
Urolithin B (**11**)	−3.80	−3.81

## Data Availability

The data are presented in the main text and as supplementary material.

## Supplementary Materials

The following are available online at https://www.mdpi.com/article/10.3390/life12030417/s1, Figure S1. 5CITEP docked to active site of HIV-1 IN (green) overlayed with 5CITEP crystal structure (gray) (PDB:1QS4); Figure S2: Best scoring docking pose of compound **1** (orange) into the active site of HIV-1 IN; Figure S3: Best scoring docking pose of compound **2** (orange) into the active site of HIV-1 IN; Figure S4: Best scoring docking pose of compound **3** (orange) into the active site of HIV-1 IN; Figure S2: Best scoring docking pose of compound **4** (orange) into the active site of HIV-1 IN; Figure S3: Best scoring docking pose of compound **6** (orange) into the active site of HIV-1 IN; Figure S4: Best scoring docking pose of compound **9** (orange) into the active site of HIV-1 IN; Figure S7. Best scoring docking pose of compound **9** (orange) into the active site of HIV-1 IN. Figure S8: Best scoring docking pose of compound 10 (orange) into the active site of HIV-1 IN; Figure S5: Best scoring docking pose of compound **11** (orange) into the active site of HIV-1 IN; Figure S6. Dose-response curve on HIV-1 Integrase for known inhibitor Sodium Azide. Figure S9. Best scoring docking pose of compound 11 (orange) into the active site of HIV-1 IN. Figure S10. Dose-response curve on HIV-1 Integrase for known inhibitor Sodium Azide. Table S1. Calculated free energy of ligands docked with HIV-1 Integrase (rigid receptor) and Table S2. Calculated free energy of ligands docked with HIV-1 Integrase (Induced fit).
