# Supplementary material for: HIV-1 Integrase Inhibitory Effects of Major Compounds Present in CareVid™: An Anti-HIV Multi-Herbal Remedy
Source: Life (Basel). 2022 Mar 12;12(3):417. doi: 10.3390/life12030417 (PMC8950597; doi:10.3390/life12030417)
Supplement: Supplementary file 1 [file life-12-00417-s001.zip › life-1624642-supplementary.pdf]

life

Supporting Information

HIV-1 Integrase Inhibitory Effects of Major Compounds Present in CareVid™: An Anti-HIV Multi-Herbal Remedy

Winnie Rotich,[1] Eduard Mas-Claret,[2] Nicholas Sadgrove,[2] Anastasia Guantai,[3] Guillermo F. Padilla-González,[2] Moses K Langat[2,*]

[1]Sigowet-Soin Sub-County Hospital, Sondu-Kapsoit Road, Sigowet, Kericho County, PO Box 112 Kericho - 20200, Kenya 1; rotichwinnie890@gmail.com
[2]Royal Botanic Gardens Kew, Kew Green, Richmond, Surrey, TW9 3AE, UK 2; e.mas-claret@kew.org; n.sadgrove@kew.org; m.langat@kew.org
[3]Department of Pharmacology and Pharmacognosy, School of Pharmacy, University of Nairobi, PO Box 19676-00202, Nairobi 2; anguantai@yahoo.com
* Correspondence: ; Tel.: (+442083325365)

1. Docking studies on HIV Integrase

The docking studies were performed on MOE2015 software package HIV-1 IN catalytic domain complexed with inhibitor 5CITEP (PDB ID: 1QS4). The protein was prepared by first removing all water molecules. Then, missing side chains were reconstructed, hydrogens were added, and the structures were protonated. The ligands were energy minimised using molecular mechanics forcefield MMFF94x. To validate the docking protocol used, known inhibitor 5CITEP was removed from their corresponding binding pockets and redocked. The Root Mean Square Deviation (RMSD) value from the known co-crystallized conformation was 0.8212 Å. The compounds were docked using MOE2015 with triangle matcher, scoring by London dG, 100 poses as placement method and rigid receptor, GBVI/WSA dG 5 poses as refinement method in all targets, and the method was then repeated using induced fit as refinement method. Both methods were repeated in three independent runs. The lowest scoring affinity pose in each ligand was used to study the ligand interactions.

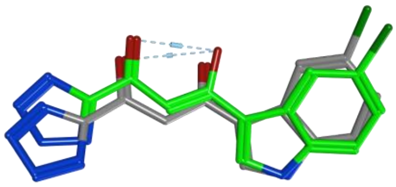

Figure S1. 5CITEP docked to active site of HIV-1 IN (green) overlayed with 5CITEP crystal strucutre (gray) (PDB:1QS4)

Table S1. Calculated free energy of ligands docked with HIV-1 Integrase (rigid receptor).

Compound	S	rmsd_refine	E_conf	E_place	E_score1	E_refine	E_score2
1	-4.33824	1.217022	-31.9376	-35.2415	-7.37015	-12.0489	-4.33824
2	-5.67268	2.574627	73.19921	-17.1664	-11.0831	-21.1705	-5.67268
3	-4.24199	1.738137	48.36356	-36.7374	-11.8061	-10.6566	-4.24199
4	-4.74533	1.467851	31.17932	-39.5445	-11.1879	-14.4274	-4.74533
6	-4.33275	1.499589	22.94607	-40.3402	-6.07212	-11.0736	-4.33275
9	-4.15551	5.758557	43.08601	-50.502	-10.5275	-16.0497	-4.15551
10	-3.94674	1.901121	24.7924	-21.3989	-7.04319	-15.6901	-3.94674
11	-3.79995	1.704051	45.79031	-3.0133	-6.54222	-14.8098	-3.79995
5CITEP	-4.41645	2.454062	37.04565	-36.9484	-11.473	-19.6001	-4.41645

Table S2. Calculated free energy of ligands docked with HIV-1 Integrase (Induced fit).

Compound	S	rmsd_refine	E_conf	E_place	E_score1	E_refine	E_score2
1	-4.21754	1.800322	-36.3754	-54.6017	-8.03353	-12.0317	-4.21754
2	-5.9361	1.910161	78.34987	-4.72883	-8.65753	-18.8714	-5.9361
3	-4.25378	2.775576	48.36929	14.70679	-11.4421	-10.6718	-4.25378
4	-4.95961	1.466584	35.56976	-37.9644	-8.88857	-15.2665	-4.95961
6	-4.21399	5.033751	24.47195	0.451038	-7.29145	-8.3912	-4.21399
9	-4.59876	5.126516	44.09461	-33.2565	-8.91852	-22.2392	-4.59876
10	-3.91398	2.876594	24.63301	5.420929	-7.18071	-14.9126	-3.91398
11	-3.80698	3.702139	45.55625	-2.90278	-6.2273	-15.1249	-3.80698
5CITEP	-4.18417	1.511891	36.07636	-37.1659	-9.58071	-14.5913	-4.18417

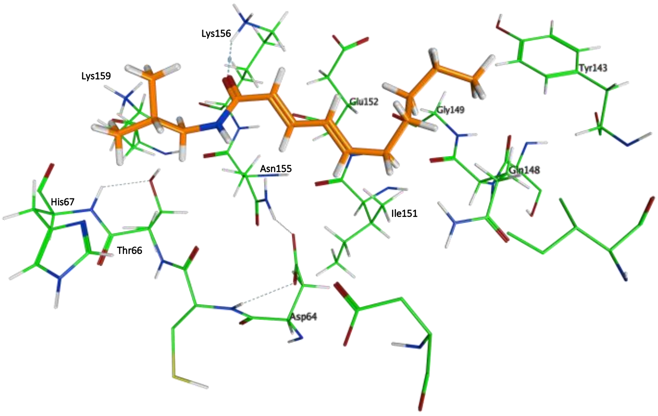

Figure S2: Best scoring docking pose of compound **1** (orange) into the active site of HIV-1 IN.

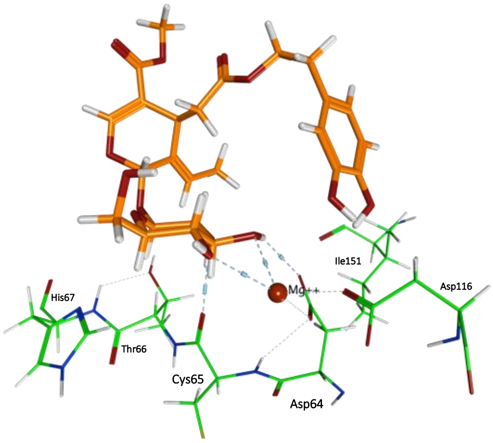

Figure S3: Best scoring docking pose of compound **2** (orange) into the active site of HIV-1 IN.

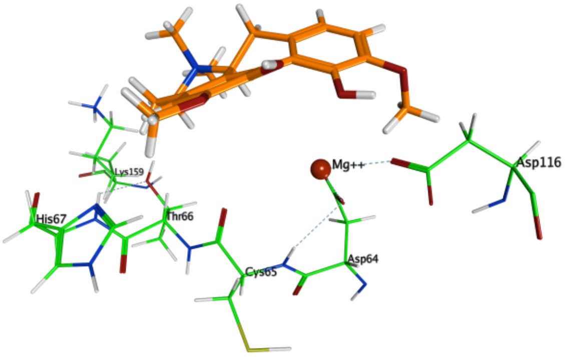

Figure S4: Best scoring docking pose of compound **3** (orange) into the active site of HIV-1 IN.

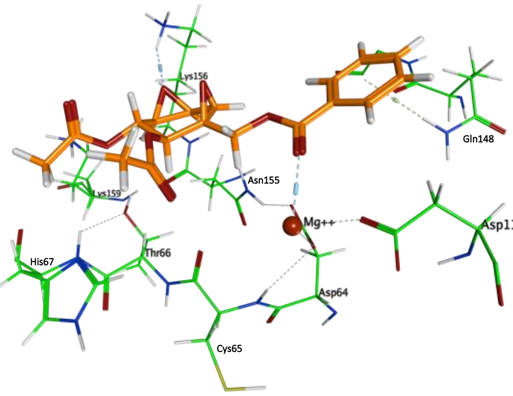

Figure S5: Best scoring docking pose of compound **4** (orange) into the active site of HIV-1 IN.

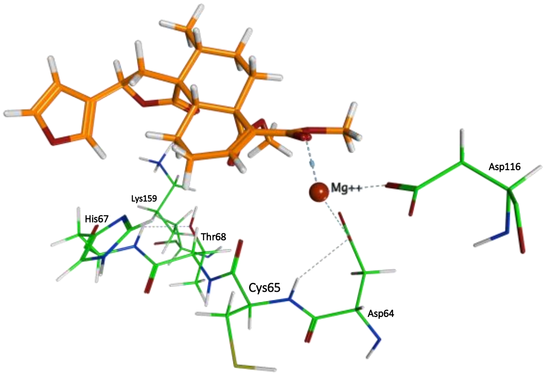

Figure S6: Best scoring docking pose of compound **6** (orange) into the active site of HIV-1 IN.

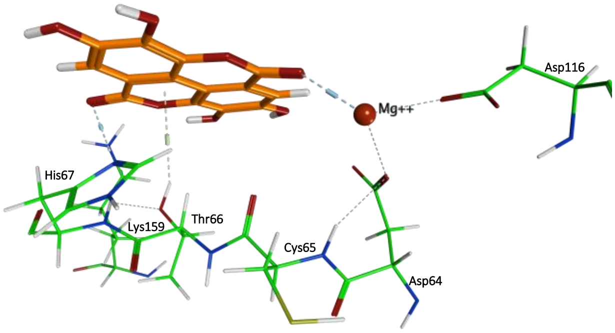

Figure S7: Best scoring docking pose of compound **9** (orange) into the active site of HIV-1 IN.

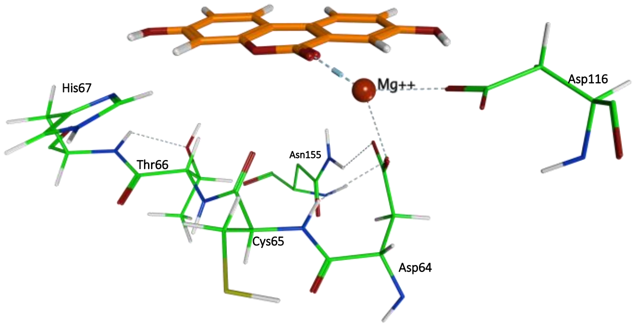

Figure S8: Best scoring docking pose of compound **10** (orange) into the active site of HIV-1 IN.

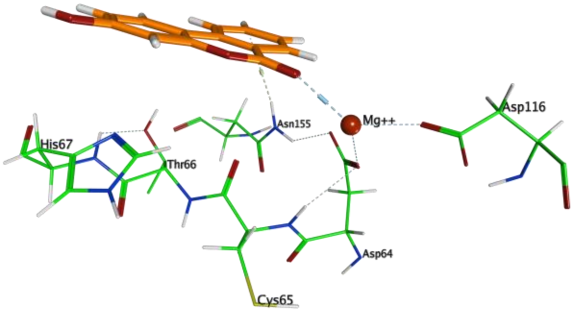

Figure S9: Best scoring docking pose of compound **11** (orange) into the active site of HIV-1 IN.

2. In vitro anti-HIV-1 Integrase assay

The XpressBio HIV-1 Integrase Assay Kit (EZ-1700, Kit Lot K1029), provided by Express Biotech International, was used to measure the inhibitory effects of the different compounds on HIV-1 IN activity following the manufacturer's instructions. Briefly, Streptavidin coated 96-well plates were coated with a double-stranded HIV-1 LTR U5 donor substrate (DS) oligonucleotide containing an end-labelled biotin. Full-length recombinant HIV-1 IN protein was then loaded onto the DS DNA substrate. Compounds and sodium azide (positive control) were added to the enzyme reaction and then a different double-stranded target substrate (TS) DNA containing a 3′-end modification was added to the reaction mixture. The HIV-1 IN cleaves the terminal two bases from the exposed 3′-end of the HIV-1 LTR DS DNA and then catalyses a strand-transfer recombination reaction to integrate the DS DNA into the TS DNA. The products of the reaction were detected calorimetrically using an HRP-labelled antibody directed against the TS 3′-end modification and the absorbance due to the HRP antibody-TMB peroxidase substrate reaction was measured at 450 nm. Compounds were prepared by dissolving to a concentration of 20 mg/mL in dimethyl sulfoxide and diluting in nuclease free water to double the desired starting concentration. A final concentration of 25 µg/mL was used for all compounds. For sodium azide, a concentration range of 0.05% – 1.5% was used to calculate its IC50 value (Figure 1S). All samples were assayed in triplicate and the averages are provided as results.

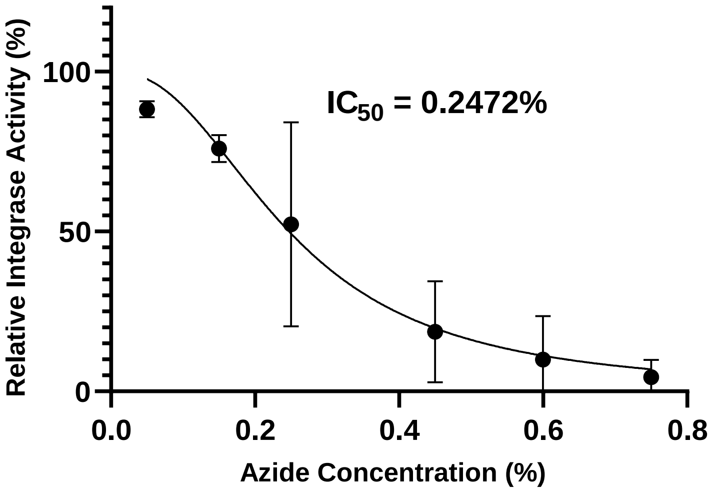

Figure S10. Dose-response curve on HIV-1 Integrase for known inhibitor Sodium Azide
